# Synergistic effects of AAGL and anti-PD-1 on hepatocellular carcinoma through lymphocyte recruitment to the liver

**DOI:** 10.20892/j.issn.2095-3941.2020.0278

**Published:** 2021-03-12

**Authors:** Xiangdong Ye, Xueqing Wang, Wenhui Yu, Qing Yang, Yan Li, Yanxia Jin, Yanting Su, Jiaqi Song, Bo Xu, Hui Sun

**Affiliations:** 1 College of Life Sciences, Wuhan University, Wuhan 430071, China; 2 Department of Biochemistry and Molecular Biology, Institute of Biomedical research, College of Basic Medicine, Hubei University of Medicine, Shiyan 442000, China; 3 Hubei Key Laboratory of Edible Wild Plants Conservation and Utilization, Hubei Normal University, Huangshi 435002, China; 4 Hubei Province key Laboratory of Allergy and Immunology; Wuhan University, Wuhan 430071, China

**Keywords:** Hepatocellular carcinoma, AAGL, anti-PD-1, immunotherapy, lymphocyte infiltration

## Abstract

**Objective:**

Therapy for hepatocellular carcinoma (HCC) is a major challenge, and targeted therapies provide only a modest benefit in terms of overall survival. Treatment with antibodies to programmed cell death protein 1 (PD-1)/PD-L1 can restore the functions of tumor-infiltrating T cells in HCC and has shown clinical efficacy in 20% of patients with advanced HCC. Novel approaches are urgently needed to treat HCC and to augment the efficacy of immunotherapy.

**Methods:**

Tumor-bearing mice were treated with *Agrocybe aegerita* galectin (AAGL) alone or in combination with anti-PD-1, and the tumor sizes and lifespans of mice were determined. Transcriptome analysis, cytokine analysis, flow cytometry analysis of the number and proportion of immune cell subsets in the liver and spleen, and molecular and cellular analyses of tumors were used to define the underlying mechanisms.

**Results:**

AAGL significantly inhibited the growth of liver tumors in a dose-dependent manner. Furthermore, AAGL increased the expression of multiple cytokines and chemokines in tumor-bearing mouse livers; this effect was associated with the activation and migration of T cells and macrophages, in agreement with the *in vitro* results. Importantly, the aggregation of T cells and macrophages induced by AAGL in tumor-bearing mouse livers clearly enhanced the response to PD-1 blockade immunotherapy.

**Conclusions:**

The results showed that AAGL induced the activation and migration of lymphocytes to the liver, and that the combination of AAGL and anti-PD-1 may be a promising strategy for HCC treatment.

## Introduction

Primary liver cancer, a disease with etiologic and geographic diversity, is the third leading cause of cancer-related death and the fifth most common tumor worldwide; hepatocellular carcinoma (HCC) accounts for most of these cases^1^. Globally, the number of deaths from HCC is close to the number of new patients diagnosed^2^. Because the disease is generally diagnosed in advanced stages, the overall 5-year survival rate is only 15%–17%^3^. Currently, the standard of care for first-line treatment of advanced HCC involves 2 multikinase inhibitors (sorafenib and lenvatinib); in patients who fail first-line treatment with these drugs, the second-line treatment involves multikinase inhibitors (regorafenib and cabozantinib)^4–7^.

Recent studies have demonstrated the potential of immunotherapy in the treatment of liver cancer and other malignancies^8,9^. In particular, blockade of programmed cell death protein 1 (PD-1) checkpoint cell surface receptors on T cells is a promising approach, although the response rates are relatively low, at ~19% in liver cancer^10^. Beyond the high heterogeneity of tumor cells in patients and the differential expression of PD-1/PD-L1 in the tumor microenvironment, the infiltration of immune cells, such as effector T cells, into the tumor microenvironment is another important factor^11^. Correlative studies have shown that expression of both PD-L1 and PD-1 in tumors is significantly correlated with HCC stage, local recurrence rate, and poor prognosis^12,13^. Similarly, the frequency of intratumoral or circulating PD-1^+^ CD8^+^ T cells correlates with HCC progression and postoperative recurrence^14^. Mariathasan^15^ has found that TGF-β is highly expressed in tumor areas that lack CD8^+^ T cell infiltration, and therapeutic co-administration of TGF-β-blocking and anti-PD-L1 antibodies facilitates T cell penetration into the tumor center, and elicits vigorous anti-tumor immunity and tumor regression. Hamada^16^ has also reported that TIL-present tumor immunity in the microenvironment subtypes of colorectal cancer are associated with better responsiveness to cancer immunotherapy, and low CD4^+^ and CD8^+^ TIL counts have also been found to predict extremely poor HCC-specific survival^17^. Therefore, efficient combinatorial strategies are needed to enhance the efficacy of PD-1/PD-L1 blockade therapy in HCC.

Lectins are carbohydrate-binding proteins that are ubiquitously expressed in viruses, bacteria, fungi, animals, and plants. Lectins contain at least one carbohydrate recognition domain, which binds carbohydrates reversibly without structural alterations, and can agglutinate cells or precipitate polysaccharides and glycoconjugates^18,19^. Many lectins have been reported to exhibit anti-tumor effects through apoptosis and autophagy. For example, Concanavalin A (ConA) directly causes tumor cell death in an autophagic manner^20^, and *Sclerotiumrolfsii* lectin inhibits the growth of human breast cancer cells through apoptosis^21^. Furthermore, some lectins exhibit immune activation activity, such as ConA, which induces hepatitis in mice through triggering natural killer (NK) T cells and subsequently activating CD4+ T cells^22–24^.

AAGL, consisting of 2 identical 15.8-kDa subunits, is a galectin purified from *Agrocybe aegerita*, an edible mushroom that is well known for its nutritional and medicinal value^25^, and has been studied for many years in our laboratory. Previous studies have reported that AAGL at concentrations above 2.0 mg/kg induces liver injury in a dose-dependent manner^26^. Additionally, AAGL induces the death of a variety of tumor cell lines (including S180 and HeLa cells) though both apoptosis and autophagy, in a manner dependent on its carbohydrate-binding activity^27^. In this study, we report that AAGL significantly inhibits liver tumor growth and prolongs the lifetimes of tumor-bearing mice by activating lymphocytes, including macrophages and T cells, and stimulating them to secrete chemokines, which in turn promote T cell infiltration into liver tumors. In combination AAGL and PD-1 blockade therapy, AAGL significantly improved the survival rate of anti-PD-1-treated tumor-bearing mice. Our results indicated that AAGL, which promotes lymphocyte infiltration, is a novel potential ancillary drug for augmenting the response to immunotherapy.

## Materials and methods

### Ethical approval

After approval was obtained from the Institutional Review Board (Approval No. WDSKY0201503), and a specific request was made to the independent ethical committee of Wuhan University, all experimental protocols for the animal studies were approved by the Ethics Committee for Animal Experimentation of Wuhan University and were in accordance with the institutional guidelines.

### Cell lines

H22, HepG2, and RAW264.7 cells were obtained from the China Center for Type Culture Collection at Wuhan University. H22 cells were cultured in RPMI medium 1640 basic (Gibco) supplemented with 10% FBS (SeraPro, S601S-500) and antibiotics (100 mg/L streptomycin and 1 × 10^5^ U/L penicillin) in a humidified atmosphere (5% CO_2_, 37 °C). Both HepG2 and RAW264.7 cell lines were maintained in DMEM containing 10% FBS and antibiotics in a humidified atmosphere.

### Purification of AAGL

Lectin AAGL was isolated and purified from the fungus *Agrocybe aegerita* as described in Sun et al.^25^, and the purified AAGL was stored at −20 °C.

### Isolation and culture of mouse primary hepatocytes

Mouse hepatocytes were isolated with a two-step collagenase perfusion technique as described in Li Wan-Chun et al.^28^.

### Mice and tumor models

Male Balb/c mice (18–20 g) were purchased from Hunan SJA Laboratory Animal Co., Ltd [quality certification number: SCXK (Xiang) 2011-0003]. The research was conducted according to protocols approved by the Animal Ethics Committee of Wuhan University, and all animal experiments were performed in the Laboratory Animal Center of the College of the Life Sciences, Wuhan University (Approval No.: WDSKY0201503). H22 ascites tumor cells were collected from mice and diluted with 0.01 M PBS (pH 7.4) to a final concentration of 6 × 10^7^ cells/mL. The hepatic carcinoma model was successfully established by injection of H22 cells (3.5 × 10^5^ cells in 5 μL) into the distal ends of the spleens of anesthetized mice (20 μL/20 g, 1% pentobarbital sodium in 0.01 M PBS). The mice bearing liver tumors were randomly distributed into 4 groups (*n* = 10): control group, AAGL group, anti-PD-1 group, and AAGL plus anti-PD-1 group. From the 6th day after tumor cell inoculation, PD-1 blockade was accomplished by administration of 200 μg of anti-PD-1 (clone: J43, BioXCell) through intraperitoneal injection twice every other day. The mice were injected with 1.5 mg/kg AAGL intravenously 3 times every other day from the 5th day to the 9th day (or 200 μL PBS). In the AAGL plus anti-PD-1 group, the mice were treated AAGL and anti-PD-1 in a combined treatment. One week after the last AAGL administration, all mice were euthanized by carbon dioxide asphyxiation, and the anti-tumor effects were assessed. The liver index was calculated as follows: liver weight/body weight.

### Transcriptome data analysis

Seven days after the splenic inoculation of H22 cells in Balb/c mice, the tumor-bearing mice were distributed into 3 groups: a 1.5 mg/kg AAGL treated group (*n* = 6), a 3.0 mg/kg AAGL treated group (*n* = 6), and a control group (*n* = 6). All tumor-bearing mice were sacrificed at 6 h after treatment with AAGL, and the whole liver tissue RNA was extracted and mixed in the same manner in every group. Sequencing was performed with a BGISEQ-500 instrument by the Wuhan Genomic Institution (www.genomics.org.cn, BGI, Shenzhen, China). Clean reads (**Supplementary Table S1**) were mapped to the reference genome and genes available in the Mouse GRC m38/mm10 reference genome. For gene expression analysis, the matched reads were calculated and then normalized to RPKM with RESM software. The significance of differentially expressed genes (DEGs) was confirmed with the BGI bioinformatics service by using a combination of the absolute value of log_2_(fold change) ≥ 1 and FDR ≤ 0.001. Analysis of the pathways and biological cascades involving the immune-related DEGs was performed on the PANTHER website (www.pantherdb.org/). The heatmap of DEGs was constructed in R software.

### Adoptive transplantation experiments

Splenocytes were isolated from healthy Balb/c mouse spleens in a sterile environment, and the red blood cells were lysed and washed 3 times with saline and labeled with 1.25 μM carboxyfluorescein diacetate succinimidyl ester (CFSE) at a splenocyte concentration of 5 × 10^7^ cells/mL. Then 2 × 10^7^ CFSE^+^ splenocytes were injected in 200 μL of saline into H22 tumor-bearing mice* via* the tail vein. Sixteen hours after the splenocyte transplantation, mice in the experimental group were administered 1.5 mg/kg AAGL through the tail vein, and 12 h later, all spleens and livers in the tumor-bearing mice were harvested, and cytometry analysis of the distribution of CFSE^+^ splenocyte subsets was performed.

### Flow cytometry analysis

Twelve hours after treatment with AAGL, after liver perfusion, mouse MNCs were isolated from the tumor-bearing mouse livers and spleens and subjected to FACS analysis as previously described. The splenic lymphocytes and T cells (480024, Biolegend) were isolated 3 h after treatment with AAGL *in vitro* and subjected to FACS analysis. The following mAbs were used: APC hamster anti-mouse CD3e (clone 145-2C11) and FITC rat anti-mouse CD8a (clone 53-6.7) from BD Biosciences (China); and PE rat anti-mouse CD4 (CL012PE), CD8a (CL168PE), CD335 (NKP46, 137604), F4/80 (CL8940PE), and IL-2 (503808), APC anti-mouse CD11b (101212), CD49b (103516), TNF-α (506308), and PC5.5 anti-mouse IFN-γ (505822) from Biolegend (Beijing, China).

### Quantitative real-time PCR

The mRNA levels of CCL2, CCL3, CCL5, CXCL9, CXCL10, IL15, IL6, IL10, Arg-1, TGF-β, and the reference gene GAPDH were measured with real-time PCR (Bio-Rad, CFX Connect™). Liver total RNA was extracted with TRIzol reagent (Invitrogen, USA) according to the manufacturer’s instructions. The cDNAs were synthesized from 1 μg of the total RNA preparation with Moloney murine leukemia virus reverse transcriptase (Promega). Quantitative real-time PCR analysis was performed with Thunderbird SYBR qPCR mix (Toyobo, Osaka, Japan) in a 10 μL reaction system. The specific quantitative PCR primers for genes are listed in **Supplementary Table S3**.

### Western blot analysis

Total protein from liver tissue was extracted and quantified with a bicinchoninic acid protein concentration assay kit (Thermo Scientific) and then separated by 12% SDS/PAGE with a Bio-Rad electrophoresis system. Blotted membranes were incubated for 1 h with blocking solution (Tris-buffered saline/0.1% Tween 20, TBST) containing 5% skim milk (w/v) at room temperature. Subsequently, the membrane was incubated overnight at 4 °C or 1 h at room temperature with primary antibody [anti-granzyme B (catalog: A2557), anti-perforin (catalog: A0093), TRAIL (catalog: A12064), and anti-Caspase-3 (catalog: A19654) polyclonal antibody from Abclonal (Wuhan, China); and anti-Caspase-8 polyclonal antibody (catalog: 13423-1-AP) and anti-GAPDH monoclonal antibody (catalog: 60004-1-Ig) from Proteintech (Wuhan, China)]. After being washed, the membrane was incubated 1 h with 1:10,000 diluted HRP-conjugated polyclonal goat anti-rabbit IgG (H+L) or HRP-conjugated polyclonal goat anti-mouse IgG (H+L) antibodies (Proteintech, China). The membranes were washed, and the immunoblot was then developed with an ECL chemiluminescence detection kit (Thermo Scientific) according to the manufacturer’s instructions. To normalize for protein loading, GAPDH was used, and the protein expression levels were calculated relative to the value of GAPDH.

### Immunohistochemistry analysis

Tumor-bearing mouse livers were fixed in 4% paraformaldehyde for 24 h, embedded in paraffin, sectioned, stained with hematoxylin and eosin, and processed for immunohistochemistry analysis. After deparaffinization and antigen retrieval, slides were incubated with primary antibodies to CD3 (GB13014, Servicebio, 1:100), CD4 (GB13064, Servicebio, 1:1,000), CD8 (GB11068, Servicebio, 1:1,500), and CD68 (GB11067, Servicebio, 1:1,000), followed by goat anti-rabbit secondary antibodies labeled with HRP (GB23303, Servicebio, 1:200). Sections were incubated with DAB substrate (DAKO, K5007) and counterstained with hematoxylin. Finally, sections were scanned with panoramic MIDI (3D HISTECH) and analyzed in CaseViewer software.

### Analysis of liver function

As liver function indicators, alanine aminotransferase (ALT) and aspartate aminotransferase (AST) were determined with commercial kits (Jiancheng, Nanjing, China) according to the manufacturer’s instructions.

### Statistical analysis

Statistical analysis was performed in GraphPad prism software. Two-tailed Student’s t test was applied for calculating statistical probability. Data were considered to be statistically significant when the *P* value was equal to or less than 0.05. Differences in survival were evaluated with a log-rank test.

## Results

### AAGL efficiently inhibited liver tumor cell growth *in vitro* and *in vivo*

**Figure 1:**
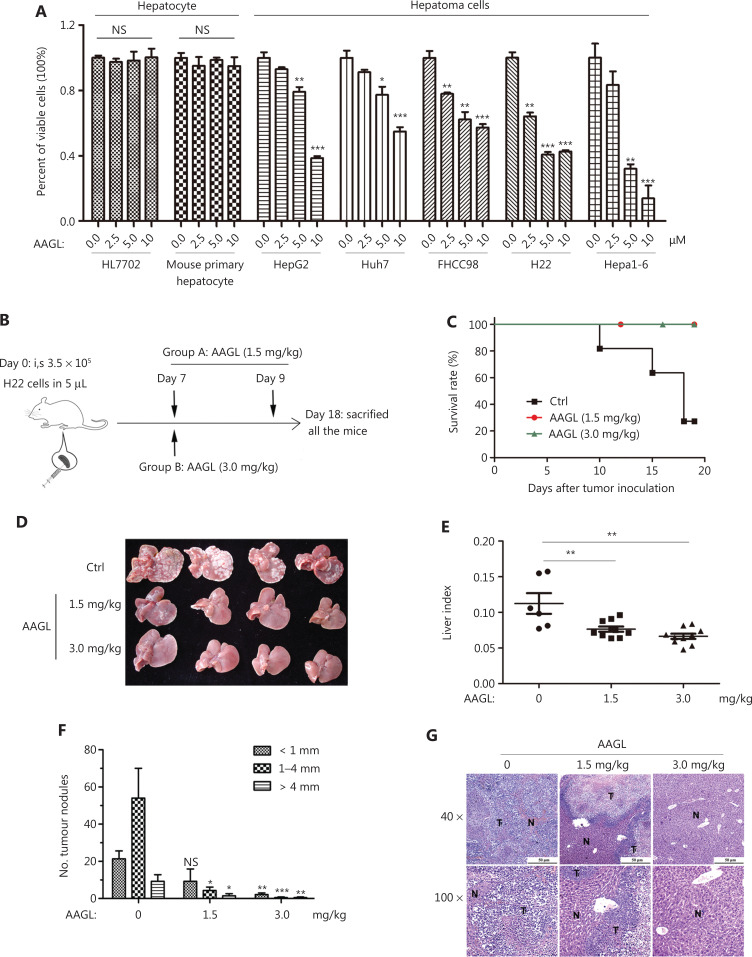
AAGL significantly inhibited liver tumor cell growth *in vitro* and *in vivo*. (A) AAGL inhibited the growth of 5 hepatoma cell lines (HepG2, Huh7, FHCC98, H22, and Hepa1-6) but not normal hepatocytes (HL7702 and mouse primary hepatocytes) *in vitro*. Cells were treated with AAGL (0, 2.5, 5.0, 10, or 20 μM) for 12 h, and cell activity was detected with CCK8 Kits. The experiments were repeated 3 times and yielded the same results. (B) The workflow for the establishment and AAGL treatment of the H22 tumor-bearing mouse model. Intra-splenic inoculation of 3.5 × 10^5^ H22 cells in 5 μL established tumor nodule formation in the livers of Balb/c mice. AAGL (1.5 mg/kg) was administered twice on day 7 and day 9 in group A (*n* = 10); group B (*n* = 10) received 3.0 mg/kg once on day 7 by intravenous injection. The control group was injected with an equal volume of PBS. All mice were sacrificed on day 18, and the livers were obtained. (C) AAGL prolonged the survival of tumor-bearing mice (*n* = 10). The experiments were repeated 3 times and yielded the same results. (D–F) AAGL inhibited tumor nodule formation in Balb/c mice (D), and tumor burdens were calculated by liver index (E, liver index = liver weight/body weight); (F) statistical analysis was performed on the number of tumor nodules on the surface of the whole liver. *P < 0.05, **P < 0.01 and ***P < 0.001 *vs*. control group. (G) H&E staining (top layer 40×, bottom layer 100×) of AAGL-treated and untreated mouse livers. T represents tumor, and N represents normal tissues.

The anti-tumor activity of AAGL on 5 hepatoma cell lines and 2 types of normal hepatocytes were investigated *in vitro*. AAGL significantly inhibited the proliferation of the 5 hepatoma cell lines in a dose dependent manner, but not the normal hepatocyte cell line HL7702 and mouse primary hepatocytes (**Figure 1A**). To investigate the effect of AAGL on the growth of liver tumors *in vivo*, we constructed a diffusely distributed multinodular HCC mouse model by injecting H22 tumor cells into the spleen. On the 5th day, white tumor nodules were observed on the surfaces of the mouse liver. Then 1.5 mg/kg or 3.0 mg/kg AAGL was administered twice every other day starting from the 7th day. All mice were sacrificed at day 18 after tumor inoculation (**Figure 1B**). The lifespan of AAGL-treated mice was markedly longer than that of the control mice (**Figure 1C**). All mouse livers were removed for tumor nodule calculation, liver index calculation, and pathological slice examination. As shown in **Figure 1D**, the numbers of tumor nodules on the liver surfaces in the AAGL-treated group were significantly lower than those in the control group, and the effect was dose dependent. The liver index was significantly lower in mice treated with AAGL than in the control mice (**Figure 1E**), thus indicating that liver tumor growth was inhibited in the AAGL-treated group.

Further statistical results showed that the numbers of tumor nodules with diameters of 1–4 mm and greater than 4 mm were both significantly lower than those in the control group, but the number of tumor nodules smaller than 1 mm was not clearly different between the 1.5 mg/kg AAGL group and the control group. However, the 3.0 mg/kg AAGL group had substantially fewer tumor nodules than the control group (**Figure 1F**). Furthermore, the hematoxylin-eosin staining results in liver tissue showed that the tumor sizes were also smaller in the AAGL-treated groups than the control, and the effect was dose dependent (**Figure 1G**). These data indicate that AAGL significantly inhibits liver tumor growth *in vivo* and *in vitro*, and may be a potential therapeutic drug for treating HCC.

### AAGL upregulates the expression levels of most immune-related genes in tumor-bearing mouse livers

**Figure 2:**
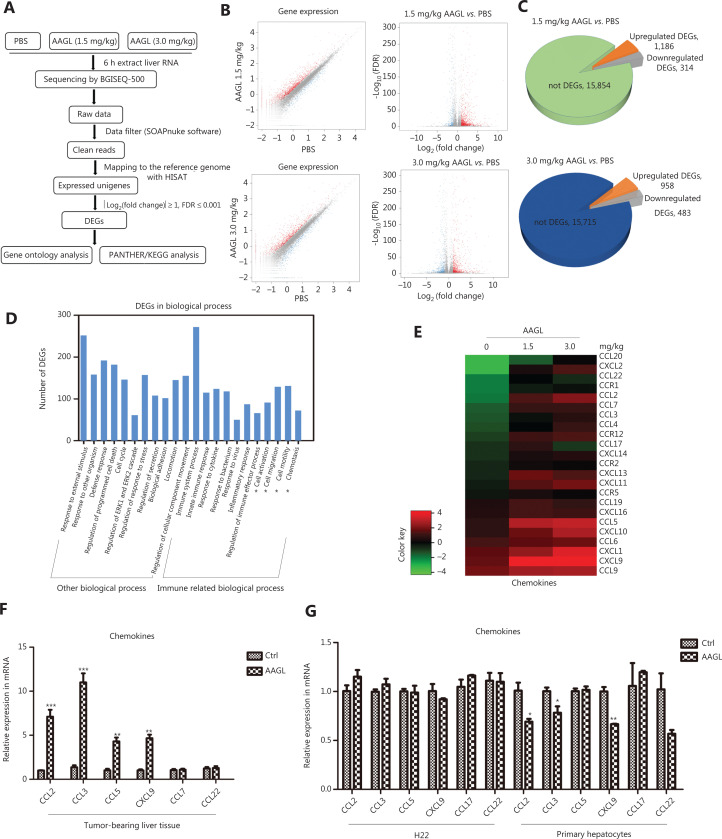
AAGL regulated the expression of immune-related genes in tumor-bearing mouse livers. (A) Pipeline of transcriptomic data analysis. (B) Expressed genes were analyzed with R (version 3.4.0) *via* scatter plots (left), and volcano plots (right). (C) Pie chart of total expressed genes. (D) Analysis of the most enriched biological processes associated with DEGs between the 1.5 mg/kg AAGL treatment group and PBS group, according to the PANTHER website. (E) Heatmap analysis of DEGs associated with chemokines induced by 1.5 mg/kg and 3.0 mg/kg AAGL in tumor-bearing mouse livers. DEGs were analyzed with the PANTHER website, and the value of log_4_ (RPKM) of DEGs was analyzed in R software. (F) Quantitative PCR analysis of CCL2, CCL5, CCL3, CXCL9, CCL17, and CCL22 mRNA expression levels in 1.5 mg/kg AAGL-treated and untreated tumor-bearing mouse liver tissues, and mRNA expression of chemokines in mouse primary hepatocytes and H22 cells after treatment with 0.625 μM AAGL for 3 h *in vitro* (G). Expression was normalized to GAPDH and is shown as fold change relative to PBS-treated mice. Samples were analyzed in triplicate and are reported as the mean ± SEM. *P < 0.05, **P < 0.01 and ***P < 0.001 *vs*. control group. The experiments were repeated 3 times and yielded the same results.

In the occurrence and development of HCC, the liver immune environments change significantly. To investigate the mechanism of the anti-tumor effect induced by AAGL in a diffusely distributed multinodular HCC mouse model, we performed transcriptome sequencing to study the expression levels of genes associated with the immune system in tumor-bearing mouse livers in the AAGL treated and control groups. First, tumor-bearing mouse livers were harvested at 6 h after treatment with AAGL or PBS, and high-quality RNA-seq data are shown in **Supplementary Table S1**. Then, the DEGs were determined with Hierarchical Indexing for Spliced Alignment of Transcripts (HISAT), and GO annotation and PANTHER analyses were performed (**Figure 2A**). For the AAGL-treated group compared with the control group, the gene expression patterns are shown in **Figure 2B**, and the up- and down-regulated DEGs are shown in **Figure 2C**. The GO annotation analysis of 1,500 DEGs (1.5 mg/kg AAGL *vs.* PBS) and 1,441 DEGs (3.0 mg/kg AAGL *vs.* PBS) involved similar biological processes and molecular functions (**Supplementary Figure S2**). The most enriched biological processes associated with the DEGs on the list were immune system processes, particularly multiple cell chemotaxis, cell migration, and cell activation (**Figure 2D**; details in **Table 1**).

**Table 1:** Summary of select biological processes of immune associated DEGs involved in cell migration, chemotaxis, and activation in H22 tumor-bearing mouse liver RNA-seq

Biological process	AAGL 1.5 mg/kg *vs.* PBS			AAGL 3.0 mg/kg *vs.* PBS		
	Gene number	Raw *P* value	FDR	Gene number	Raw *P* value	FDR
Immune system process	272	3.72E-28	1.44E-24	315	5.03E-48	8.22E-44
Cell migration	129	8.30E-22	5.14E-19	110	1.86E-15	3.89E-13
Leukocyte migration	56	1.76E-19	9.42E-17	52	1.07E-17	3.26E-15
Lymphocyte migration	20	1.52E-09	1.20E-07	18	2.17E-08	1.51E-06
Neutrophil migration	31	2.43E-13	4.09E-11	32	1.84E-14	3.13E-12
Chemotaxis	72	4.09E-12	5.33E-10	78	1.24E-15	2.71E-13
Leukocyte chemotaxis	38	7.32E-14	1.35E-11	40	1.19E-15	2.67E-13
Lymphocyte chemotaxis	13	3.86E-06	1.88E-04	14	5.21E-07	2.80E-05
Neutrophil chemotaxis	29	5.73E-13	8.71E-11	29	2.34E-13	3.21E-11
Cell activation	91	3.15E-15	6.97E-13	100	3.00E-20	1.13E-17
Lymphocyte activation	63	2.30E-10	2.00E-08	69	1.31E-13	1.86E-11
T activation	41	1.84E-08	1.47E-06	41	1.14E-07	6.85E-06
Macrophage activation	65	4.89E-10	3.97E-08	61	3.42E-13	4.53E-11

DEGs, differentially expressed genes; AAGL, *Agrocybe aegerita* galectin.

A heatmap analysis of the expression of immune-related DEGs involved in chemokine regulation indicated that AAGL treatment significantly increased the expression of those genes (**Figure 2E**) in a concentration-dependent manner. In addition, KEGG pathway analysis of DEGs showed that the TNF signaling pathway (ko04668) and NK cell-mediated cytotoxicity (ko04650), which coincided with cell activation and the chemokine signaling pathway (ko04062), were consistent with the cell migration and cell chemotaxis (**Supplementary Table S2 and Figure 2D**). The chemokines CCL2, CCL3, CCL5, and CXCL9, which are associated with CD4^+^ T, CD8^+^ T, and NK cell migration^29–31^, and CCL17 and CCL22, which are associated with Treg and myeloid-derived suppressor cell migration^32,33^, were selected for further quantitative PCR validation. The quantitative PCR results were consistent with the transcriptome results, showing that the expression of CCL2, CCL3, CCL5, and CXCL9, but not CCL17 or CCL22, was markedly higher in 1.5 mg/kg AAGL-treated tumor-bearing mouse livers than in those of control mice (**Figure 2F**). Given that tumor cells and parenchymal hepatic cells regulate the tumor environment by secreting cytokines and chemokines, we also detected chemokine expression in AAGL-treated H22 cells and primary hepatocytes by quantitative PCR. No distinct elevations were observed in AAGL-treated H22 cells or primary hepatocytes, as compared with the control group levels, thus indicating that AAGL significantly promotes the expression of chemokines in immune cells, but not hepatoma H22 cells and parenchymal hepatic cells (**Figure 2G**). These results also suggested that AAGL might inhibit liver tumor growth by up-regulating the expression of chemokines in liver.

### AAGL activates macrophages and T cells

**Figure 3:**
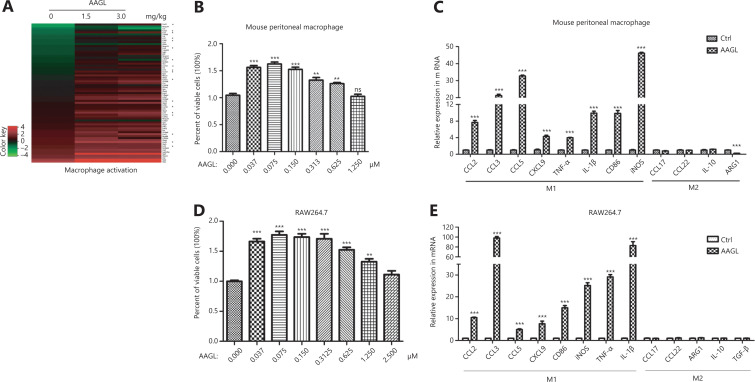
AAGL specifically promoted the activation of macrophages *in vivo* and *in vitro*. (A) Heatmap analysis of DEGs associated with macrophage activation induced by 1.5 mg/kg and 3.0 mg/kg AAGL in tumor-bearing mouse livers. (B) AAGL promotes the proliferation of mouse peritoneal macrophages at a low dose *in vitro*. The proliferation of macrophages was detected with a CCK-8 kit (*n* = 5). (C) Quantitative PCR analysis of the mRNA expression of chemokines and cytokines in mouse peritoneal macrophages after treatment with 0.625 μM AAGL for 3 h. (D) AAGL promotes the proliferation of RAW264.7 cells at a low dose of AAGL (≤1.25 μM) *in vitro*. The proliferation of RAW264.7 cells was detected with a CCK-8 kit (*n* = 5). (E) Quantitative PCR analysis of the mRNA expression of chemokines and cytokines in RAW264.7 cells after treatment with 0.625 μM AAGL for 3 h. *P < 0.05, **P < 0.01 and ***P < 0.001 *vs*. control group.

Further analysis of the DEGs involved in cell activation showed that 65 DEGs were associated with macrophage activation (**Table 1**). Heatmap analysis indicated that 61 DEGs were significantly up-regulated in a dose-dependent manner after 1.5 mg/kg and 3.0 mg/kg AAGL treatment, including 18 chemokines (labeled by asterisks) in H22 tumor-bearing mouse livers (**Figure 3A**), thus indicating that AAGL activates macrophages. An additional macrophage activation assay was performed *in vitro* to verify this conclusion. The results showed that AAGL at a low dose distinctly promoted the proliferation of mouse peritoneal macrophages (**Figure 3B**) and RAW264.7 cells (**Figure 3D**). The expression of several cytokines was analyzed by RT-PCR. The results showed that AAGL treatment distinctly increased the expression of M1 differentiation-related cytokines, including CCL2, CCL3, CCL5, CXCL9, TNF-α, IL-1β, and CD86, and the M1 marker iNOS, in both peritoneal mouse macrophages (**Figure 3C**) and RAW264.7 cells (**Figure 3E**). However, the expression of several anti-inflammatory cytokines, such as CCL17, CCL22, IL-10, and TGF-β, and the M2 marker ARG1, was not significantly changed; these results were consistent with the transcriptome data in H22 tumor-bearing mouse liver tissue. Thus, AAGL was found to activate macrophages and induce the polarization of M1 macrophages. Macrophages may be the source of the upregulated chemokines in H22 tumor-bearing mice in response to AAGL treatment.

**Figure 4:**
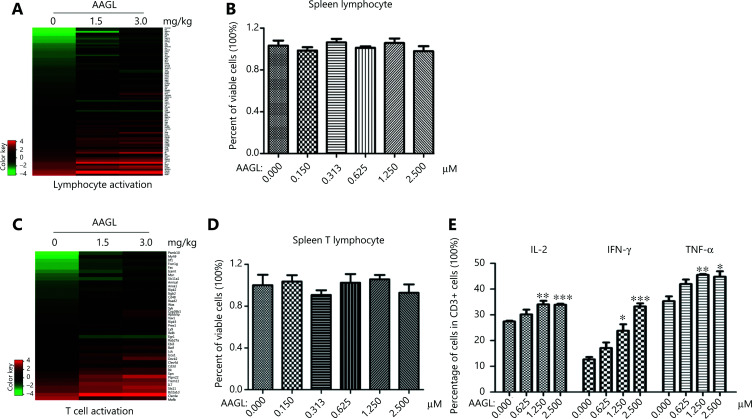
AAGL specifically promoted the activation of T cells* in vivo* and *in vitro*. (A) Heatmap analysis of DEGs associated with lymphocyte activation induced by 1.5 mg/kg and 3.0 mg/kg AAGL in tumor-bearing mouse livers. (B) AAGL did not affect the proliferation of lymphocytes *in vitro*. The proliferation of lymphocytes was detected with a CCK-8 kit. (C) Heatmap analysis of DEGs associated with T cell activation induced by 1.5 mg/kg and 3.0 mg/kg AAGL in tumor-bearing mouse livers. (D) AAGL did not affect the proliferation of splenic T cells *in vitro*. The proliferation of T cells was detected with a CCK-8 kit. (E) Flow cytometry analysis of the percentages of IL2^+^, IFN-γ^+^, and TNF-α^+^ in CD3^+^ cells from mouse splenic T lymphocytes, which changed in a dose-dependent manner *in vitro*. Samples were analyzed in triplicate on biological replicates and are reported as the mean ± SEM. *P < 0.05, **P < 0.01 and ***P < 0.001 *vs*. control group. The experiments were repeated 3 times and yielded the same results.

In addition, transcriptome analysis revealed that AAGL significantly increased the expression of cytokines associated with lymphocyte activation (**Figure 4A**), particularly T cell activation, in a dose-dependent manner (**Figure 4C**). A proliferation assay was performed, and the cytokine expression levels in lymphocytes and T cells were analyzed. As shown in **Figure 4B, 4D**, although AAGL treatment did not promote the proliferation of splenic lymphocytes and T cells, the flow cytometry results showed that the percentages of IL-2^+^, IFN-γ^+^, and TNF-α^+^ CD3^+^ T cells all distinctly increased after treatment with AAGL in a dose-dependent manner *in vitro* (**Figure 4E**). Our data indicated that AAGL activates T cells immediately and therefore may play a pivotal role in the anti-tumor effect of AAGL in our model.

### AAGL induces migration of spleen T cells and macrophages to the liver

**Figure 5:**
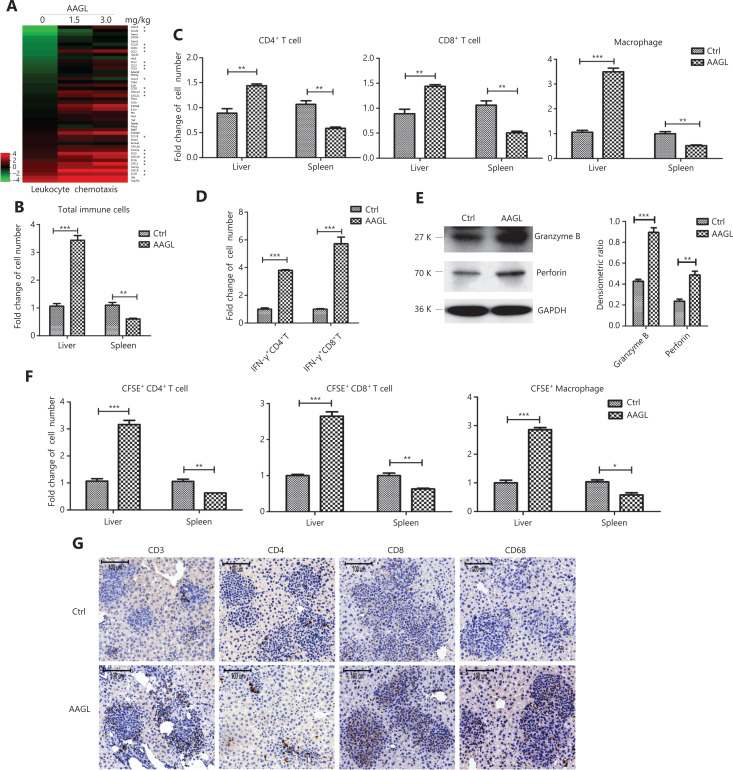
AAGL stimulated splenic T cell and macrophage infiltration into liver tumors. (A) Heatmap analysis of DEGs associated with leukocyte chemotaxis induced by 1.5 mg/kg and 3.0 mg/kg AAGL in tumor-bearing mouse livers. Chemokines are labeled with an asterisk. (B) Flow cytometry analysis of the numbers of total immune cells in the livers and spleens of tumor-bearing mice. (C) Flow cytometry analysis of immune cell subsets, including CD4^+^ T cells, CD8^+^ T cells, and macrophages, in the livers and spleens of tumor-bearing mice. A total of 1.5 mg/kg AAGL was administered on day 5 after tumor cell inoculation, and liver and spleen immune cells were isolated 12 h after treatment. CD4^+^ T cells were defined as CD3^+^ and CD4^+^ cells, CD8^+^ T cells were defined as CD3^+^ and CD8^+^ cells, and macrophages were defined as CD11b^+^ and F4/80^+^ cells. (D) Flow cytometry analysis of IFN-γ^+^ CD4^+^ T cells and IFN-γ^+^ CD4^+^ T cells in tumor-bearing mouse livers 12 h after treatment with 1.5 mg/kg AAGL. (E) Western blot analysis of the expression levels of perforin and granzyme B in liver immune cells isolated from AAGL-treated and untreated tumor-bearing mice (*n* = 3). (F) Flow cytometry analysis of the following CFSE-labeled immune cells in tumor-bearing mouse livers with splenocyte transplantation tests: CFSE^+^ CD4^+^ T cells, CFSE^+^ CD8^+^ T cells, and CFSE^+^ macrophages. Splenocytes from healthy mice were labeled with 0.1 μM CFSE, and 2 × 10^6^ cells were injected into tumor-bearing mice *via* the tail vein. AAGL at 1.5 mg/kg was given after 16 h of blood circulation and again after another 12 h, after which the CFSE^+^ splenocyte subsets in the liver were evaluated. The experiments were repeated 3 times and yielded the same results. (G) Immunohistochemistry stains (200×) of CD68 (macrophage), CD3 (total T cells), CD4, and CD8 labelling (in brown) in liver tissues of tumor-bearing mice. The immunostaining is representative of tissues from *n* = 6 mice. *P < 0.05, **P < 0.01 and ***P < 0.001 *vs*. control group.

Heatmap analysis of the DEGs involved in leukocyte chemotaxis showed that several chemokines (labeled by asterisks), accounting for 42% of leukocyte chemotaxis, were upregulated in the 2 AAGL-treated groups (**Figure 5A**). The up-regulation of chemokines in the liver suggested that AAGL may induce the infiltration of immune cells into liver tissue in H22 tumor-bearing mice. To confirm this hypothesis, we isolated immune cells from the tumor-bearing mouse livers. Flow cytometry results showed that the numbers of total immune cells increased 3.32-fold in the liver but decreased by half in the spleen after treatment with AAGL (**Figure 5B**). Further analysis indicated that the numbers of CD4^+^ T cells, CD8^+^ T cells, and macrophages in the liver significantly increased after treatment with 1.5 mg/kg AAGL (**Figure 5C**). In contrast, the numbers of CD4^+^ T cells, CD8^+^ T cells, and macrophages in the spleens of the AAGL-treated group were significantly decreased compared with the control group. Additionally, analysis of cytotoxic T cells indicated that the IFN-γ^+^ CD4^+^ T cells and IFN-γ^+^ CD8^+^ T cells increased 3.8- and 5.7-fold, respectively, in H22 tumor-bearing mice after treatment with AAGL (**Figure 5D**). The protein levels of perforin and granzyme B in lymphocytes isolated from H22 tumor-bearing mouse livers were markedly higher than those in the control group (**Figure 5E**). In the liver tissue of H22 tumor-bearing mice, the RT-PCR results showed that the mRNA expression levels of FasL and TRAIL were significantly higher in AAGL-treated mice than control mice (**Supplementary Figure S3A**). The protein levels of perforin, TRAIL, cleaved caspase-8, and caspase-3 in liver tissue increased at both 6 and 12 h after AAGL treatment (**Supplementary Figure S3B**). These data strongly suggested that AAGL effectively improved the cytotoxicity of CD4^+^ T cells and CD8^+^ T cells, in agreement with the transcriptome sequencing results (**Figure 4A**). These data indicated that AAGL may induce migration of spleen lymphocytes, including T cells and macrophages, to liver tissue and the activation of T cells in H22 tumor-bearing mice.

An adoptive transplantation of splenocytes was performed to confirm this conclusion. Spleen lymphocytes collected from healthy mice and labeled with 0.1 μM CFSE were transplanted into H22 tumor-bearing mice *via* tail vein injection after 24 h; then the mice were treated with AAGL. Flow cytometry analysis showed that in the AAGL-treated group, compared with the control group, the numbers of liver CFSE-positive CD4^+^ T cells, CD8^+^ T cells, and macrophages were 3.16-, 2.64-, and 2.85-fold higher, respectively. Moreover, the numbers of CFSE-positive CD4^+^ T cells, CD8^+^ T cells, and macrophages in the spleen were almost all half those in the control group after treatment with AAGL (**Figure 5F**). Furthermore, immunohistochemistry analysis revealed that there were more T cells, including CD4^+^ T cells, CD8^+^ T cells, and macrophages, in the tumor nodules of liver tissue in the treated mice than in the control mice (**Figure 5G**). These results indicated that AAGL treatment induces spleen immunocyte infiltration into the liver in H22 tumor-bearing mice.

### The anti-tumor effect of AAGL is T cell dependent

**Figure 6:**
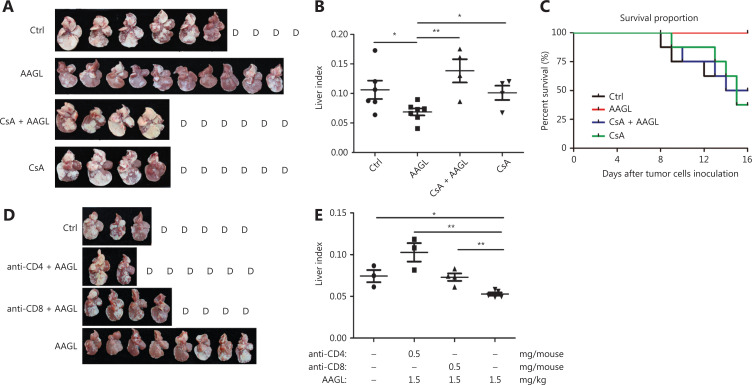
The anti-tumor effect of AAGL depended on liver T cells. (A) Cyclosporine A (CsA) eliminated the inhibition of tumor nodule formation by AAGL in Balb/c mice. AAGL (1.5 mg/kg) was administered 3 times on days 5, 7, and 9 *via* the tail vein in a 200 μL volume. A dose of 50 mg/kg CsA in 200 μL of PBS was administered by peritoneal injection half an hour before AAGL injection. All surviving tumor-bearing mice were sacrificed on day 16, and liver tissues were harvested. (B) Statistical analysis of the liver index of tumor-bearing mice after treatment with CsA and AAGL. Liver index = liver weight/body weight. (C) Statistical analysis of the survival curve of tumor-bearing mice after treatment with CsA and AAGL. (D) Both anti-CD4 and anti-CD8 abrogated the inhibition of tumor nodule formation by AAGL in Balb/c mice. A dose of 0.5 mg/kg anti-CD4/CD8 was administered by peritoneal injection 1 h before the administration of AAGL. All surviving tumor-bearing mice were sacrificed on day 16, and liver tissues were harvested. (E) Statistical analysis of the liver index of tumor-bearing mice after treatment with anti-CD4/CD8 and AAGL. *P < 0.05, **P < 0.01 and ***P < 0.001 *vs*. control group. The experiments were repeated 3 times and yielded the same results.

The above data showed that AAGL induces T cell activation and migration into liver tissue, and indicated that T cells may play a pivotal role in this process. T cell inhibitors, including cyclosporine A (CsA), anti-CD4, and anti-CD8, were used to investigate the function of T cells in our model (**Figure 6**). The results revealed that AAGL treatment significantly inhibited liver tumor growth (**Figure 6A**), decreased the liver index (**Figure 6B**), and prolonged the survival time in tumor-bearing mice compared with control mice (**Figure 6C**). However, CsA completely abrogated the anti-tumor effect of AAGL; almost all tumor nodules on the liver surface had connected; and no differences in the liver index and the survival period of tumor-bearing mice were observed between the CsA plus AAGL treatment group and the control group. There were no significant differences in tumor growth, liver index, and the survival period of tumor-bearing mice between the group treated with CsA alone and the control group (**Figure 6A–6C**). These data indicated that T cells played a pivotal role in the anti-tumor effect of AAGL. The results of blockade of CD4^+^ T cells and CD8^+^ T cells showed that both anti-CD4 and anti-CD8 completely abolished the anti-tumor effects of AAGL. Anti-CD4/anti-CD8 blockade accelerated the death of tumor-bearing mice and the tumor severity (**Figure 6D**). In addition, the liver index (**Figure 6E**) was much higher in the antibody blockade group than in the group treated with AAGL alone. These data suggested that both CD4^+^ T and CD8^+^ T cells play crucial roles in the anti-tumor effect of AAGL.

### Combination treatment with AAGL and anti-PD-L1 suppresses HCC progression

**Figure 7:**
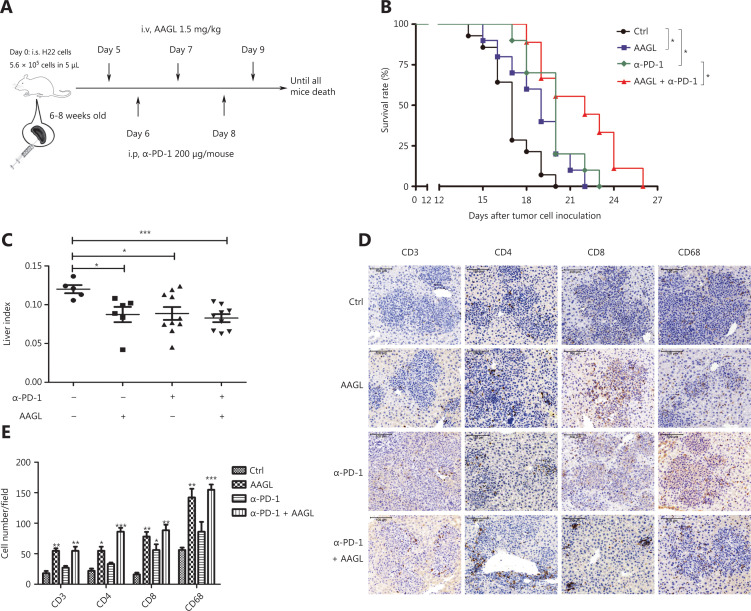
AAGL synergized with anti-PD-1, thereby enhancing the effect of tumor killing. (A) Workflow of the combination therapy of AAGL and anti-PD-1 in tumor-bearing mice (*n* = 10). AAGL at a dose of 1.5 mg/kg in 200 μL of PBS was administered 3 times (on days 5, 7, and 9), and anti-PD-1 was given at a dose of 200 μg in 100 μL of PBS per mouse by intraperitoneal injection on days 6 and 8. (B) The survival curve after combination therapy with AAGL and anti-PD-1 in H22 tumor-bearing mice. * means *P* < 0.05. (C) Statistical analysis of the liver index of combination therapy with AAGL and anti-PD-1 in H22 tumor-bearing mice (*n* = 8–10), as compared with AAGL or anti-PD-1 treatment alone. (D) Immunohistochemistry staining (200×) of macrophages (CD68), CD4^+^ T cells, and CD8^+^ T cells labeled in brown in tumor-bearing mouse livers after treatment with combination therapy, AAGL, or anti-PD-1. (E) Statistical analysis of immunohistochemistry staining (200×) of liver tissue slices, *P < 0.05, **P < 0.01 and ***P < 0.001 *vs*. control group, *n* = 4.

Topalian et al.^34^ have demonstrated that PD-1 blockade is a particularly effective immunotherapy approach in which the cumulative response rates (all doses) are 18% among patients with non-small cell lung cancer (NSCLC), 28% among patients with melanoma, and 27% among patients with renal-cell cancer. Blockade of PD-1 checkpoint cell surface receptors on T cells is a promising approach, although the response rates are relatively low (~19%) in liver cancer^10^. One reason contributing to therapy failure may be that the significantly decreased cytotoxic T cells and NK cells in solid tumors are partly a result of the strong immunosuppressive tumor microenvironment^35,36^. Our data demonstrated that AAGL treatment induced cytotoxic T cell recruitment in the liver. Therefore, we further investigated the effects of combination treatment with AAGL and anti-PD-1 on H22 tumor-bearing mice. The workflow is shown in **Figure 7A**. H22 tumor-bearing mice were separated into 3 groups: an AAGL group, an anti-PD-1 group, and a combination (anti-PD-1 and AAGL) treatment group. The AAGL group mice were treated with 1.5 mg/kg AAGL on the 5th, 7th, and 9th days by tail vein injection; the anti-PD-1 group mice were treated with 200 μg/mouse anti-PD-1 on day 6 by intraperitoneal injection; and the combination treatment group mice were treated with both anti-PD-1 and AAGL. Survival rate analysis revealed that mice treated with AAGL and anti-PD-1 had longer lifespans than those of the controls, and the mice receiving combination treatment had significantly longer lifespans than those of mice treated with AAGL or anti-PD-1 alone (**Figure 7B**). The liver indexes were analyzed and are shown in **Figure 7C**. Compared with control mice, the mice receiving AAGL, anti-PD-1, and combination treatment showed significantly lower liver indexes. The cell numbers of macrophages, CD4^+^ T cells, and CD8^+^ T cells were calculated on the basis of immunohistochemistry analysis of liver tissue slices. Anti-PD-1 treatment, compared with the control resulted in only slightly higher CD4^+^ T and macrophage cell numbers in the liver tissue. AAGL treatment significantly increased the lymphocyte numbers in liver tissue. In the combination group, the numbers of macrophages, CD4^+^ T cells, and CD8^+^ T cells were higher than those in the anti-PD-1 and control groups (**Figure 7D, 7E**). These data suggested that AAGL has a synergistic effect on anti-PD-1 treatment in H22 tumor-bearing mice through recruiting lymphocytes to the liver tumor microenvironment. A combination treatment with AAGL and anti-PD-1 may therefore be a promising strategy for liver cancer treatment.

## Discussion

Many lectins are known to be T cell mitogens and to have anti-tumor and antiviral activity. Related studies have focused on directing cytotoxicity to a variety of tumor cells by inducing autophagy and apoptosis, such as with *Polygonatum cyrtonema* lectin and ConA^37,38^. Investigations of the effects of lectins on the immune system have also focused on mitogenic activity and pro-inflammatory activity, by stimulating macrophages and T lymphocytes in variety of inflammatory diseases, such as hepatitis and colitis^39,40^. The effects of lectins on the tumor immune system have rarely been reported. Although AAGL induces apoptosis and autophagy in tumor cells when the dose exceeds 1.25 μM *in vitro* (**Supplementary Figure S1**), the dose of AAGL used in this study was 0.47 μM (calculated according to the assumption that each mouse has 2 mL blood); this concentration was much lower than 1.25 μM and significantly inhibited the tumor growth *in vivo*. The treatment with CsA or anti-CD4/anti-CD8 completely abolished the anti-tumor effect of AAGL. Together, our results indicated that the immune system plays a crucial role in our model. Here, we uncovered a new role of a lectin (AAGL) in recruiting immune cells into the liver, thus contributing to a significant inhibitory effect on tumor growth.

The results of the redistribution of CFSE-labeled splenic cells after treatment with AAGL showed that the numbers of CD4^+^ T cells and CD8^+^ T cells decreased in the peripheral blood (**Supplementary Figure S4E, S4F**). These findings indicated that AAGL also recruits lymphocytes in the peripheral blood to the liver. The increased number of immune cells in the liver may come not only from the spleen but also from the peripheral blood and other lymphoid organs. We also found that the number and the cytotoxicity of NK cells (**Supplementary Figure S4A–S4C**) in the liver significantly increased after treatment with AAGL, in agreement with the transcriptome sequencing results (**Supplementary Table S2**). Interleukin 15 was upregulated in AAGL-treated tumor-bearing mouse livers; this cytokine plays a critical role in the survival, maturation, proliferation, and activation of NK cells^41,42^ (**Supplementary Figure S4D**). AAGL not only recruited lymphocytes and macrophages from the spleen into the liver but also increased the recruitment of other immune cells from peripheral blood and other organs to the liver.

In tissues, macrophages are activated in a dynamic response to combinations of stimuli, thereby acquiring specialized functional phenotypes^43^. M1 macrophages have cytotoxic and anti-tumoral properties, and M2 macrophages are generally more prone to immunoregulatory and tumor promoting activities^44^. In our model, the transcriptome analysis of liver tissue indicated that AAGL significantly induced the activation of the M1 phenotype and resulted in the secretion of a series of pro-inflammatory cytokines and chemokines, including CXCL9, CCL2, 3, 5, and IL-15. These results were additionally validated in the livers of tumor-bearing mice, peritoneal macrophages, and RAW264.7 cells, but not in H22 cells and hepatocytes. Kupffer cells are one of most abundant immune cells in the liver, accounting for 80% of all macrophages in the body. Our data indicated that macrophages were an important source of chemokines in the AAGL treated mice. Although the precise mechanism of activation of macrophages induced by AAGL in this model is unclear, previous studies have indicated that AAGL interacts with Mincle directly, thus activating macrophages^45^.

Accumulating findings from studies of cytokine function are consistent with our results. CXCL9 has been reported to direct the migration and stimulate the adhesion of activated T cells and NK cells by binding and activating CXCR3, CCL5, CCL3, and CCL4^46,47^. These chemokines not only play critical roles in recruiting lymphocytes into the liver but also may directly affect the functions of T cells. CXCL9/MIG and CXCL10 have been reported to inhibit tumor growth and metastasis of NSCLC and hemangiosarcoma^48,49^. However, M2-related cytokines, including CCL2, CCL17, and CCL22, which have been reported to recruit myeloid-derived suppressor cells and enhance the aggregation of regulatory T cells^32,50^, were not altered in the livers of the AAGL-treated mice. Therefore, we speculate that the pro-inflammatory effects of chemokines and cytokines secreted by the recruited M1 phenotype macrophages increased the cytotoxicity of T cells and NK cells in H22 tumor-bearing mouse livers.

Previous studies have found that a high dose of AAGL (>2.0 mg/kg) promotes excessive accumulation and activation of T, NK, and NKT cells in the liver, thus leading to severe liver injury in mice^26^. However, at a dose of 1.5 mg/kg, no significant changes in serum ALT and AST were observed in H22 tumor-bearing mice after treatment with AAGL (**Supplementary Figure S3C**); therefore, AAGL exhibits few liver toxicological effects on tumor-bearing mice at a dose of 1.5 mg/kg. We also found that AAGL induced the death of a variety of tumor cells, but not the human normal liver cell line HL7702 or primary hepatocytes (**Supplementary Figure S1B**). These data suggested that AAGL shows a great difference in activity between healthy and tumor-bearing mice. We presume that those active lymphocytes recruited to the liver by AAGL might induce hepatitis in healthy mice and induce tumor toxicity in hepatoma-bearing mice. Thus, AAGL could be exploited as novel potential anti-HCC agent.

The FDA approval of anti-CTLA4 and anti-PD-1 treatments has led to a new era in the treatment of cancer through ‘immune checkpoint blockade’. Immune checkpoint inhibitors, such as ipilimumab (monoclonal antibody to CTLA4) and nivolumab (monoclonal antibody to PD-1), have demonstrated survival benefits in multiple tumor types, including melanoma, non-small cell lung cancer, and renal cell carcinoma. Indeed, PD-L1 is expressed in 82% of HCC specimens (immunohistochemistry) and shows particularly higher expression levels in hepatitis B-positive patients^51^. However, a phase I/II study of nivolumab in patients with advanced HCC has demonstrated only a 19% response rate (including 5% complete responses)^10^. Tumor-infiltrating CD4^+^, CD8^+^ T cells, and NK cells in patients with HCC have been found to be functionally compromised, and the low CD4^+^ and CD8^+^ TIL counts have been found to predict extremely poor HCC-specific survival^17^. Because of the impressive anti-tumor effect but low response rate achieved with checkpoint blockade therapy, novel strategies are needed to improve the response rate. Increasing the tumor infiltrating lymphocyte count may be one such strategy. Here, we report that AAGL treatment promotes T cell, NK cell, and macrophage infiltration into the liver and extends the lifetime of tumor-bearing mice. Our results also indicated that AAGL significantly increases the cytotoxicity of CD8^+^ T cells and NK cells.

Combination therapy with AAGL and anti-PD-1 further prolonged the lifespan and resulted in cell numbers of T cells and macrophages in the livers of H22 tumor-bearing mice exceeding those observed after AAGL or anti-PD-1 therapy alone. These data indicated that AAGL stimulates splenic lymphocytes to infiltrate into the liver and thus may contribute to overcoming the barrier of low lymphocyte count in the tumor microenvironment and to enhancing the anti-PD-1 therapy effect in liver cancer. Therefore, PD-1 blockade combined with AAGL might be a promising strategy for liver tumor therapy that can compensate for the deficiency of immune cells in the liver tumor microenvironment. Together, our data indicated that combination therapy with AAGL and anti-PD-1 has a strong, synergetic anti-tumor effect.

In summary, our study reveals a new immunomodulatory activity of AAGL, which inhibits liver tumor growth by stimulating macrophages to secrete chemokines specifically recruiting T cells, NK cells, and macrophages into liver tumors. Combination therapy with anti-PD-1 and AAGL elicited stronger anti-tumor immune responses and resulted in longer lifetimes, thus overcoming the deficiency of immune cells in the tumor microenvironment in response to PD-1 blockade alone. Our findings suggest the potential of combination therapy with immune checkpoint therapy for patients with HCC.
