# Supplementary material for: Synergistic effects of AAGL and anti-PD-1 on hepatocellular carcinoma through lymphocyte recruitment to the liver
Source: Cancer Biol Med. 2021 Mar 12;18(4):1092–108. doi: 10.20892/j.issn.2095-3941.2020.0278 (PMC8610148; doi:10.20892/j.issn.2095-3941.2020.0278)
Supplement: Supplementary file 1 [file cbm-18-1092-s001.pdf]

Supplementary materials

Table S1 Statistical results of genome mapping and the quantity of reads in the liver transcriptomes of tumor bearing mice

Sample	Total raw reads (M)	Total clean reads (M)	Clean reads ratio (%)	Clean reads Q20 (%)	Clean reads Q30 (%)	Total mapping ratio (%)	Total gene number
PBS	22.99	22.88	99.52	97.15	87.01	85.54	16,225
AAGL 1.5 mg/kg	23.05	22.94	99.52	97.19	86.96	85.22	16,710
AAGL 3.0 mg/kg	23.09	22.96	99.41	97.2	87.02	86.93	16,294

Transcriptome sequencing was performed with a BGISEQ-500 instrument by the Wuhan Genomic Institution (www.genomics.org.cn, BGI, Shenzhen, China), and the numbers of clean reads all exceeded 22 M in the 3 groups; the ratio of clean reads exceeded 99%. The mapping rate of clean reads to mouse reference genomes exceeded 85%. These data suggested that the depth of sequencing and the quality of clean reads were qualified and suitable for further analysis.

Table S2 The most enriched pathways associated with DEGs in AAGL-treated mice compared with control mice

Pathway	AAGL 1.5 mg/kg *vs.* PBS		AAGL 3.0 mg/kg *vs.* PBS	
	Gene number	*P* value	Gene number	*P* value
TNF signaling pathway (ko04668)	32	1.05E-07	34	3.02E-09
Chemokine signaling pathway (ko04062)	41	4.06E-07	38	2.62E-06
Natural killer cell mediated cytotoxicity (ko04650)	30	2.74E-06	33	3.33E-08
Osteoclast differentiation (ko04380)	32	6.51E-06	42	1.50E-11
Toll-like receptor signaling pathway (ko04620)	26	3.07E-05	27	5.04E-06
Apoptosis (ko04210)	35	3.50E-05	30	8.81E-04
Cell adhesion molecules (CAMs) (ko04514)	36	4.65E-05	46	7.13E-10
Phagosome (ko04145)	40	5.57E-05	48	1.36E-08
Cytokine-cytokine receptor interaction (ko04060)	43	6.24E-05	52	7.69E-09
NF-kappa B signaling pathway (ko04064)	21	1.65E-04	23	9.54E-06
Pathways in cancer (ko05200)	41	6.55E-04	53	4.87E-03

Pathways of DEGs involved in tumor-bearing mouse livers were analyzed with the KEGG pathway database. The most enriched pathways are listed and included TNF signaling pathway, chemokine signaling pathway, NK cell mediated cytotoxicity, and apoptosis.

Table S3 mRNA primers

Gene name	Forward primer (5′-3′)	Reverse primer (5′-3′)
CCL2	TTAAAAACCTGGATCGGAACCAA	GCATTAGCTTCAGATTTACGGGT
CCL3	CCAAGTCTTCTCAGCGCCATA	GATGAATTGGCGTGGAATCTTC
CCL5	ACTCCCTGCTGCTTTGCCTTAC	GCGGTTCCTTCGAGTGACA
CXCL9	ATGCACGATGCTCCTGCA	AGGTCTTTGAGGGATTTGTAGTGG
CXCL10	GGAGTGAAGCCACGCACAC	ATGGAGAGAGGCTCTCTGCTGT
IL15	AGAGGCCAACTGGATAGATGT	AGAGCACGTTTCTTACTGTTTCA
IL10	CTGTGAAAAACAAGAGCAAGGC	GAAGCTTCTGTTGGCTCCC
IL6	AGCCAGAGTCCTTCAGAGAGATACAG	GGTCTTGGTCCTTAGCCACTCC
IL-1β	GCAACTGTTCCTGAACTCAACT	ATCTTTTGGGGTCCGTCAACT
TGF-β	CTCCCGTGGCTTCTAGTGC	GCCTTAGTTTGGACAGGATCTG
ARG1	CTCCAAGCCAAAGTCCTTAGAG	GGAGCTGTCATTAGGGACATCA
FasL	ATGGTTCTGGTGGCTCTGGT	GTTTAGGGGCTGGTTGTTGC
TRAIL	AAGTGTGTCTCCAAAACGG	AATGCACAGAGTTCGCACT
GAPDH	AGGTCGGTGTGAACGGATTTG	TGTAGACCATGTAGTTGAGGTCA

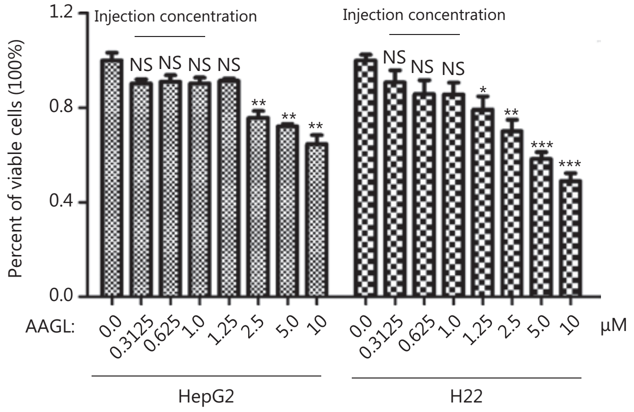

Figure S1 AAGL inhibited the proliferation of hepatoma cells in a dose-dependent manner. AAGL inhibited the proliferation of HepG2 and H22 cells in a dose-dependent manner. High doses of AAGL (≥1.25 μM) significantly inhibited HepG2 and H22 proliferation *in vitro*. Treatment with 0–1.0 μM AAGL for 12 h *in vitro* did not influence the proliferation of HepG2 and H22 at a low dose of AAGL (≤1.25 μM), The proliferation of HepG2 and H22 was detected with a CCK-8 kit. *P < 0.05, **P < 0.01 and ***P < 0.001 *vs.* control group.

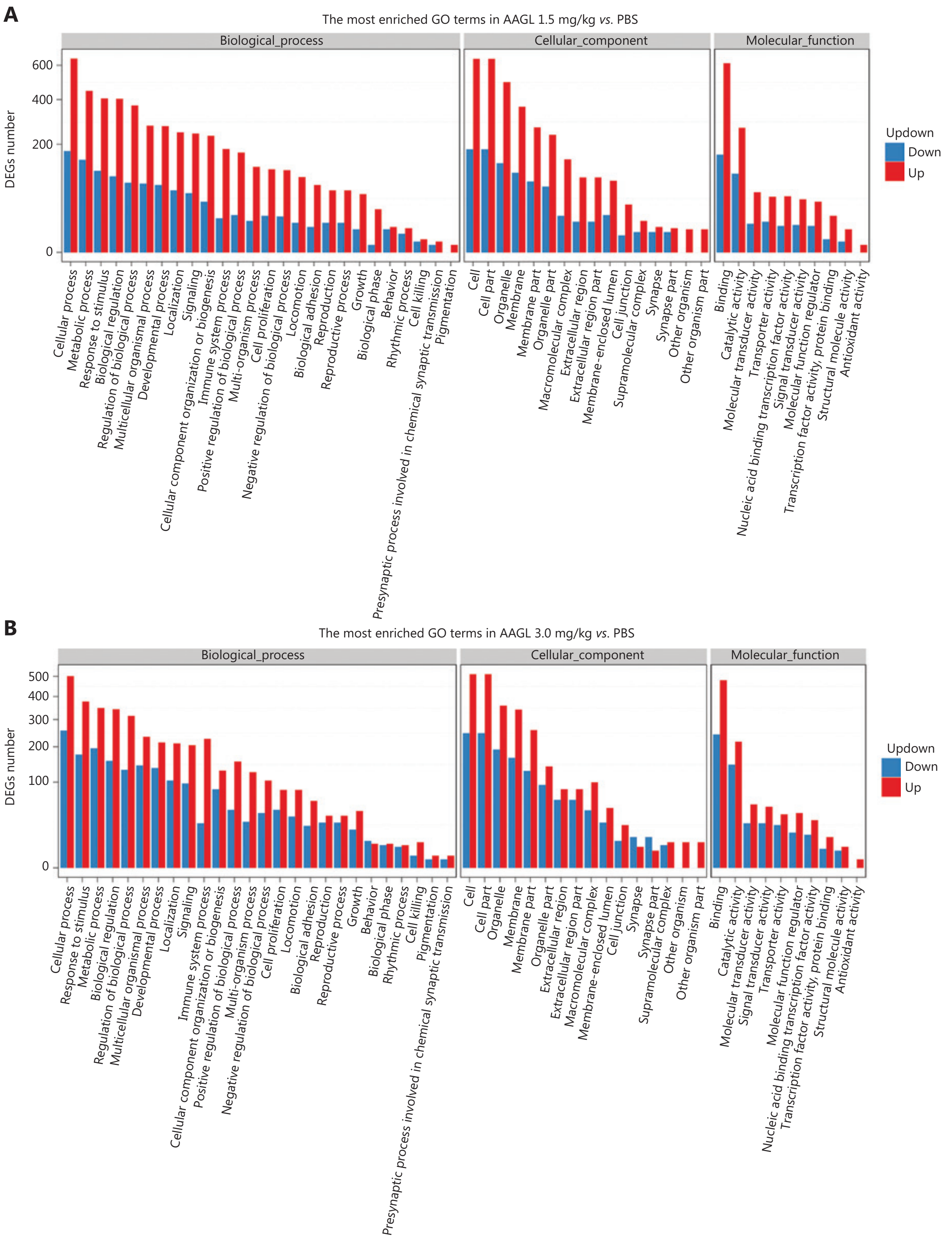

Figure S2 Two doses of AAGL resulted in similar gene expression patterns in the livers of H22 tumor-bearing mice and control mice. (A) Statistics on the number of GO functional genes in the 1.5 mg/kg treatment group compared with the control group. (B) Statistics on the number of GO functional genes in the 3.0 mg/kg treatment group compared with the control group.

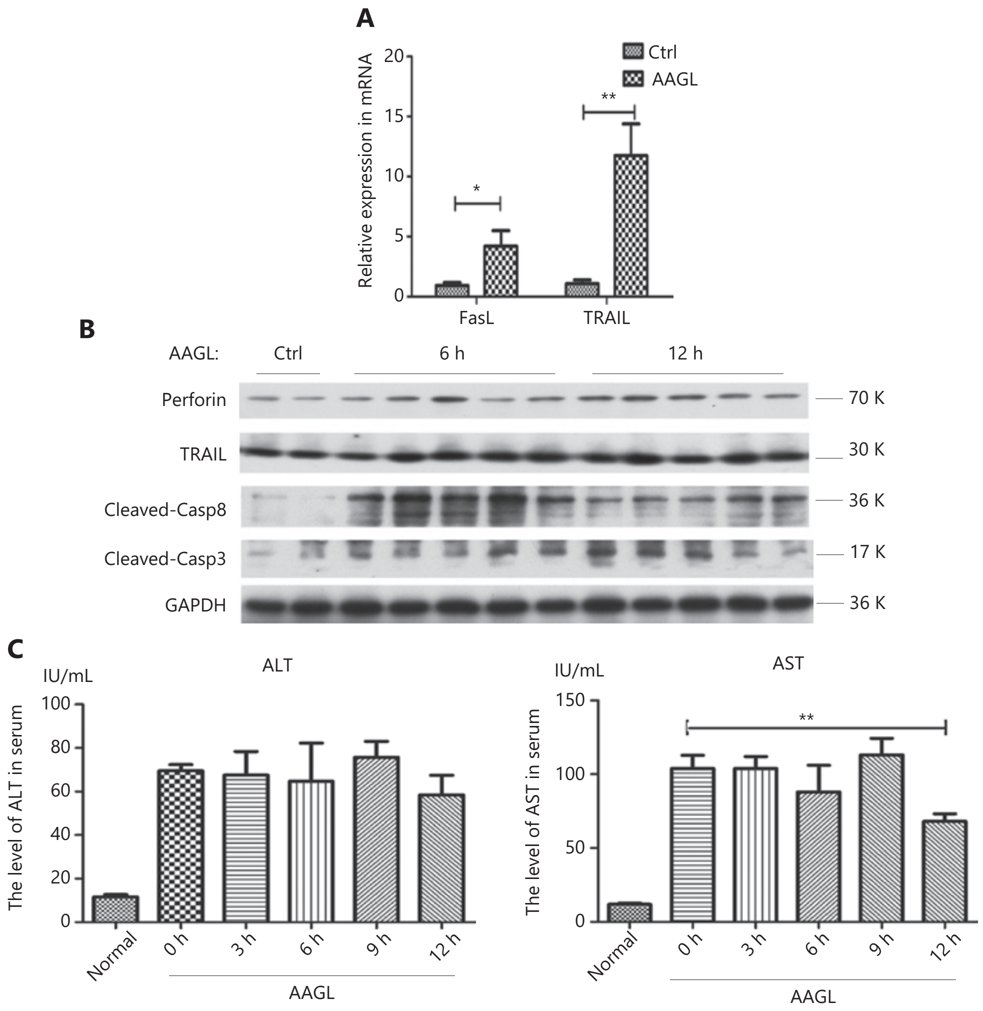

Figure S3 AAGL inhibited tumor growth by increasing the cytotoxic effects of liver T cells. (A) Quantitative PCR analysis of the death receptor associated genes FasL and TRAIL in 1.5 mg/kg AAGL treated and untreated H22 tumor-bearing mouse liver tissues. (B) Western blot analysis of perforin, TRAIL, cleaved-caspase-8, and cleaved-caspase-3 in tumor-bearing mouse livers after 6 h and 12 h of 1.5 mg/kg AAGL treatment. GAPDH was used an internal control. (C) Effects of AAGL on the serum hepatic function parameters ALT and AST in tumor-bearing mice at different time points. Serum were collected at 3, 6, 9, and 12 h after 1.5 mg/kg AAGL treatment of tumor-bearing mice at day 5 (n = 6). *P < 0.05 and **P < 0.01 *vs*. control group.

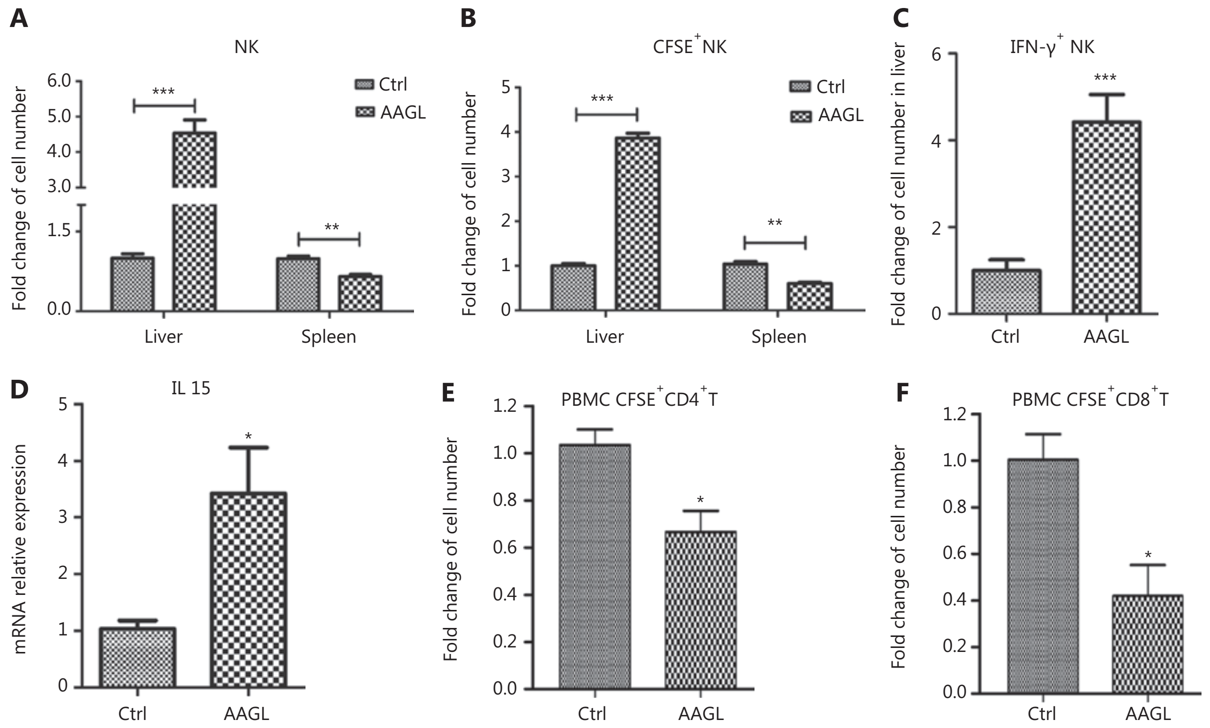

Figure S4 AAGL recruited lymphocytes to the liver and increased the cytotoxicity of liver T lymphocytes and NK cells. (A) Flow cytometry analysis of the numbers of NK cell in tumor-bearing mouse livers 12 h after the treatment with 1.5 mg/kg AAGL. (B) Flow cytometry analysis of the CFSE$^+$ NK cells in tumor-bearing mouse livers after treatment with AAGL. The spleen cells were collected from healthy mice and labeled with 0.1 μM CFSE, then transferred to tumor-bearing mice by tail vein injection. Administration of AAGL was performed at 16 h after immune cell adoptive transfer. (C) Flow cytometry analysis of the numbers of IFNγ$^+$ NK cells in tumor-bearing mouse livers 12 h after treatment with 1.5 mg/kg AAGL. Flow cytometry analysis of CFSE$^+$ CD4$^+$ T cells. (D) Quantitative PCR analysis of the gene IL-15 in 1.5 mg/kg AAGL treated and untreated H22 tumor-bearing mouse liver tissues. (E–F) Flow cytometry analysis of the CFSE$^+$ CD4$^+$ T cells (E) and CFSE$^+$ CD8$^+$ T cells (F) from peripheral blood in tumor-bearing mice after treatment with AAGL. *$P < 0.05$, **$P < 0.01$ and ***$P < 0.001$ *vs*. control group. The experiments were repeated 3 times and yielded the same results.
